# Inverse modeling unveils governing law of mechano-chemical dynamics of epithelial migration

**DOI:** 10.1371/journal.pcbi.1013854

**Published:** 2025-12-29

**Authors:** Yuto Kikuchi, Yoshifumi Asakura, Kazuhiro Aoki, Yohei Kondo, Honda Naoki

**Affiliations:** 1 Graduate School of Integrated Sciences for Life, Hiroshima University, Higashihiroshima, Hiroshima, Japan; 2 Nagoya University, Graduate school of Medicine, Nagoya, Aichi, Japan; 3 Laboratory for Developmental Morphogeometry, RIKEN Center for Biosystems Dynamics Research (BDR), Minatojima-Minamimachi, Kobe, Hyogo, Japan; 4 Exploratory Research Center on Life and Living Systems (ExCELLS), National Institutes of Natural Sciences, Okazaki, Aichi, Japan; 5 National Institute for Basic Biology, National Institutes of Natural Sciences, Okazaki, Aichi, Japan; 6 Graduate School of Biostudies, Kyoto University, Toshidakonoe-cho, Sakyo-ku, Kyoto, Japan; 7 Center for Living Systems Information Science, Graduate School of Biostudies, Kyoto University, Toshidakonoe-cho, Sakyo-ku, Kyoto, Japan; 8 Nagoya University, Center for One Medicine Innovative Translational Research (COMIT), Nagoya, Aichi, Japan; Arizona State University, UNITED STATES OF AMERICA

## Abstract

Collective cell migration is fundamental to tissue homeostasis and underlies biological processes such as wound healing and cancer invasion. Previous work has proposed governing equations to describe how chemical and mechanical inputs regulate these movements, but the quantitative validity of such models remains to be thoroughly assessed. Here, we developed a machine-learning framework that infers the governing equation from live-cell imaging data. Applied to epithelial sheet migration driven by MAPK/ERK, our approach quantitatively predicted single-cell movement from local chemical and mechanical cues. Examination of the learned equations further indicated that cells process environmental signals by computing their spatiotemporal derivatives. Moreover, when applied to individual cells, our framework revealed cell-cell heterogeneity in the underlying migratory rules. Our framework offers a powerful tool for predictive modeling of multicellular dynamics in both physiological and pathological settings.

## Author summary

Tissues heal and cancers invade when cells move together, yet we still lack a testable account of how chemical and mechanical cues combine to steer this motion. We created a data-driven framework that learns the governing rule directly from live cell movies. Applied to epithelial sheets where MAPK/ERK waves drive collective migration, the learned rule predicts each cell’s next movement from local chemical and mechanical information. Interrogating the rule shows that cells rely on absolute signal levels and on how those signals change across space and time: gradients and rates of change. The framework also reveals cell to cell differences in these migratory rules and a clear front versus interior distinction within the same sheet. Cells at the wound edge respond mainly to the instantaneous spatial gradient, whereas interior cells are more sensitive to recent temporal changes. Finally, we used the learned rule to refine a forward model, improving its ability to reproduce observed behavior. By translating raw imaging data into predictive rules, our approach provides a general tool to connect data with mechanism in multicellular dynamics and to inform quantitative studies of tissue repair and disease.

## Introduction

Collective cell migration is a fundamental driver of tissue homeostasis and underpins a variety of biological processes, including wound healing and cancer invasion [1–7]. These coordinated movements are orchestrated by intricate biochemical networks, whose disruption is closely associated with pathogenesis [8,9]. Recent advances in live-cell imaging have enabled the comprehensive tracking of the motion of every cell within a tissue, while simultaneously visualizing intracellular signaling dynamics. This wealth of spatiotemporal data provides unprecedented opportunities to elucidate the mechanistic principles driving collective cell behaviors, a long-sought goal in biological research. To realize this ambition, here we developed a biophysics-tailored machine learning framework that bridges single-cell dynamics and tissue-scale coordination, providing a data-driven pathway to uncover the rules underlying collective cell migration. Earlier experimental work has pinpointed the mitogen-activated protein kinase (MAPK) extracellular signal-regulated kinase (ERK) as a master regulator of collective migration [10–12]. ERK is a serine/threonine kinase best known for integrating mitogenic cues to control proliferation, differentiation and oncogenic transformation [13–16], yet it has also emerged as a key node linking biochemical signaling to emergent tissue-scale motion. ERK promotes cell motility by phosphorylating cytoskeletal proteins such as myosin light chain kinase and focal adhesion kinase [17]. Furthermore, in epithelia, ERK activity propagates to neighboring cells via EGFR signaling and/or mechano-responsive pathways [18]. These intercellular interactions generate traveling waves of ERK activity within a tissue, enabling coordination of collective cell migration.

ERK-dependent control of collective behavior is remarkably conserved across vertebrates, having been observed in the zebrafish and mouse [19,20]. Among in vitro systems, wound-healing assays in Madin-Darby canine kidney (MDCK) epithelial monolayers have proved uniquely tractable for mechanistic dissection [21]. In this model, ERK activity originates at the wound edge and propagates across the cultured epithelia, whereas cells migrate toward the lesion, suggesting that motility is directed opposite to the travelling ERK wavefront. Using Förster resonance energy transfer (FRET)-based biosensors, we previously captured the spatiotemporal choreography of ERK activity waves and concomitant cell trajectories during MDCK wound closure [22,23]. Furthermore, by optogenetically imposing synthetic ERK propagation within the monolayer, we demonstrated that the kinase wave itself was sufficient to steer collective migration in situ, underscoring ERK’s causal role in coordinating tissue-scale movement.

The growing body of high-resolution spatiotemporal data on collective cell migration has led to the development of sophisticated mathematical models [22,24–27]. One of the key remaining challenges is to elucidate the causal link between single-cell motion control and emergent tissue-scale dynamics. From this perspective, we developed a hierarchical modeling approach that translates a particle-based cell-level model into a continuum tissue-level framework [28]. This study not only reproduced tissue motion driven by ERK activity waves but also proposed a plausible mechanism for single-cell motion control during epithelial wound healing.

A qualitative match between simulation outputs and observational data is insufficient to confirm that the model faithfully captures physiological cellular behavior. Moreover, the predictive capability of such mathematical models at the level of individual cell trajectories has yet to be rigorously assessed. These limitations motivate data-driven inverse approaches, wherein time-series data derived from actual cell movements are used to infer the governing mechano-chemical laws underlying their behavior. Such inverse analyses enable the extraction of biologically grounded control principles directly from phenomena—an advance that is not achievable through forward modeling alone.

We presented an inverse analysis framework that uncovers how cells integrate biochemical and mechanical signals to control their movement. Previous mathematical models in this field have often been guided by intuition from researchers and have typically assumed that cells behave as instantaneous reflexes to external cues. In reality, however, cells transform incoming biochemical and mechanical signals through complex intracellular processes before making directional movement decisions. This complexity has largely been overlooked in conventional models. To address this gap, we developed a temporally extended model combined with a biophysics-tailored machine-learning approach. By applying our framework to time-series imaging of signaling activity and cell trajectories, we directly inferred how changes in chemical cues and tissue deformation drove the motion of individual cells.

## Results

**Fig 1:**
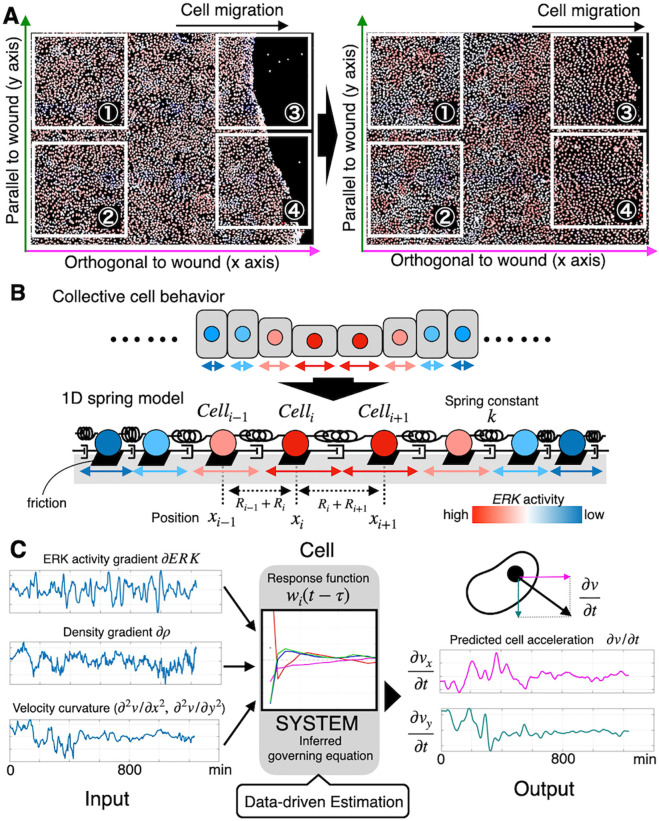
Snapshot of imaging data and framework overview. (A) Snapshots of imaging data of MDCK epithelial cells used for analysis (left: initial state/right: cells spreading into the wounded region). Red indicates high ERK activity, while blue indicates low ERK activity. Over time, cells spread into the wound area. The numbered areas represent the clipped regions used for analysis: ① Training data of interior cells, ② Test data of interior cells, ③ Training data of front cells, and ④ Test data of front cells (See S1 Fig and S1 Video for details). (B) Schematic of the one-dimensional spring-particle model used in previous studies. Cells are represented as particles connected by elastic interactions, forming the theoretical basis for our continuum model (Eq (1)). In the forward simulations (Fig 6), the data-driven response function learned in this study was incorporated into this model to evaluate improvements in its behavior. (C) Framework overview. Each cell is assumed to determine its acceleration based on the set of input information represented by the terms on the right-hand side of Eq (1). The aim of this study is to establish a data-driven framework to understand the cellular decision-making process by inferring how the time-dependent coefficients of Eq (1) are determined.

This study aimed to uncover how cells translate MAPK/ERK activity and mechanical environments into collective behavior by learning governing principles from time-series data. Specifically, we derived equations linking ERK activity waves with coordinated cell migration during wound healing (Fig 1A, S1 Fig and S1 Video). To this end, we introduced a data-driven inverse modeling framework that not only estimated model parameters but also predicted single-cell trajectories in imaging data (Fig 1B).

**Fig 2:**
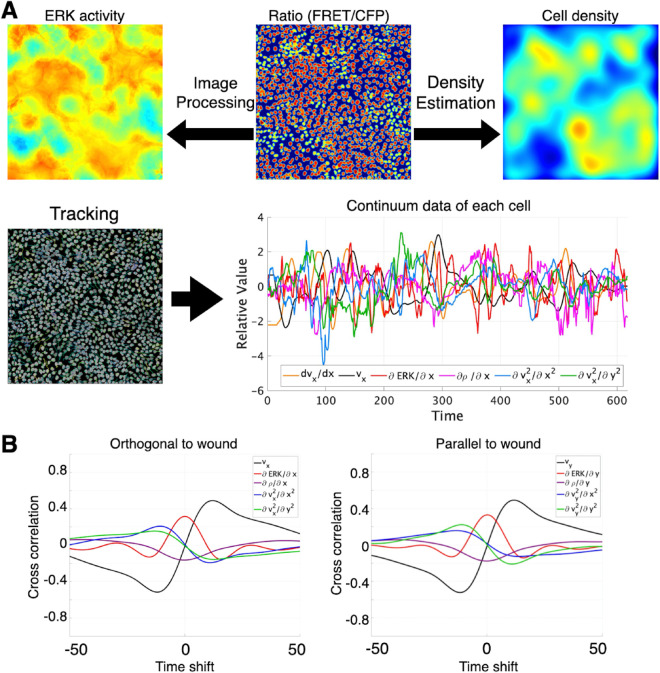
Live imaging of ERK-driven collective migration and extraction of single-cell time-series data. (A) Quantification workflow for the imaging data. Automated cell detection and tracking yield the 2-D position and ERK activity of every cell in each frame. Cell density is computed with kernel density estimation (KDE). Temporal and spatial derivatives are obtained by fitting second-order polynomials to spatio-temporal distributions of ERK activity, cell density, etc. This analysis provides cell acceleration, the spatial gradient of ERK activity, the spatial gradient of cell density, and the second spatial derivatives of cell velocity. The horizontal axis represents elapsed time in frames; images were acquired every 2 min. (B) Mean pairwise cross-correlations of the input features across cells. Correlations were computed for time lags from -100 to +100 min in both the orthogonal and parallel directions relative to the wound edge. Black, velocity; red, spatial gradient of ERK activity; purple, spatial gradient of cell density; blue, second spatial derivative of velocity along the orthogonal axis; green, second spatial derivative along the parallel axis.

As the target variable for model prediction, we quantified the acceleration of each cell at every time point based on cell tracking (Fig 2A). Since we used two-dimensional imaging data, acceleration was decomposed and quantified along two directions: orthogonal and parallel to the wound-healing axis (see Fig 1A). The input variables to model were from ERK activity and mechanical context. We introduced the local cell density, denoted by ρ(r,t), which was computed from nuclear positions using a Gaussian kernel (see Material and methods). They were also quantified for the same individual cells.

To build our regression framework to analyze the above data, we first consider the spring-mass model (Fig 1B and Material and methods). We previously demonstrated that ERK activity waves spatiotemporally regulate cell density and actomyosin-dependent mechanical responses, leading to collective migration opposite to the direction of wave propagation. Then we modeled this phenomenon by representing cells as a one-dimensional chain of particles connected by springs. In the model, ERK-dependent modulation of cell-substrate friction and the natural cell length is required to reproduce such collective migration [22,29]. Furthermore, by taking the continuum limit of this discrete model, we proposed a more analytically tractable description in terms of the cell velocity and density fields [28]. However, this model was derived from a one-dimensional discrete representation. To describe the mechanics of a two-dimensional epithelium, we therefore develop a new continuum model that incorporates the additional effects necessary for 2D tissue dynamics. When expressed with tunable free parameters as coefficients, this model can be written as follows (see Material and methods for details):

$$\frac{Dv_{x}}{Dt}≃\overset{ERK-dependent\;friction}{\overbrace{R_{0}v}}+\overset{ERKactivity}{\overbrace{R_{1}\frac{\partial ERK}{\partial x}}}+\overset{density}{\overbrace{R_{2}\frac{\partial \rho }{\partial x}}}+\overset{tissue\;viscosity}{\overbrace{R_{3}\frac{\partial^{2}v_{x}}{\partial x^{2}}+R_{4}\frac{\partial^{2}v_{x}}{\partial y^{2}}}}$$ (1)

where the coefficients *R*_0_ − *R*_4_ were inferred from the data. These coefficients are not functions of ρ but constant parameters estimated by regression. This formulation implied that cell acceleration could be approximated as a linear combination of mechanical parameters and ERK activity. This formulation was implemented using the convolutional regression model defined in Eq (3), which incorporates the temporal history of mechano-chemical inputs (see Material and methods).

In modeling realistic cell migration, it was important to recognize that the model we constructed might not fully reproduce the experimental data. To enhance the flexibility of the model, we therefore allowed the cell response to depend not only on the current input but also on its recent input history. We referred to the temporally extended coefficients that described this history-dependent response as the response functions.

### Prediction of acceleration and response function

The model used five input features: (i) cell velocity, (ii) the spatial gradient of ERK activity, (iii) the spatial gradient of cell density, and (iv,v) the second spatial derivatives of velocity along the x and y axes, respectively. Correlation analysis revealed only weak associations. Cell velocity showed a slight positive correlation with ERK activity and slight negative correlations with its own second spatial derivatives in both directions (S2 Fig). Because these pairwise correlations were small, each feature was expected to provide largely independent information to the model. Next, we computed cross correlations between the input features across time lags from 100 to –100 minutes. To assess whether anisotropy existed with respect to the wound edge, we computed cross correlations in the orthogonal and parallel directions separately (Fig 2B). This analysis confirmed that the cropped data exhibited no pronounced anisotropy.

**Fig 3:**
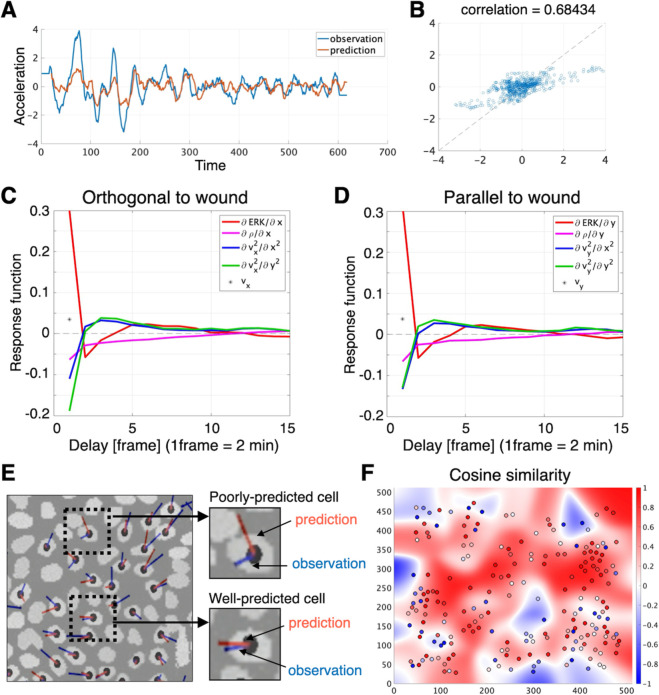
Prediction of single-cell acceleration and inference of response function. (A) Time course of acceleration for a representative cell. Blue, measured values; orange, model predictions. (B) Scatter plot of the same cell’s measured versus predicted accelerations. (C,D) Response functions learned for predicting cell accelerations orthogonal and parallel to the wound edge. For every input feature except velocity, time lags up to 20 frames (40 min) were considered; only the first 15 frames are displayed here. Black points indicate velocity; red, the spatial derivative of ERK activity; purple, the spatial derivative of cell density; blue, the second spatial derivative along the orthogonal axis; and green, the second spatial derivative along the parallel axis. All response functions converged to zero within ∼10 frames (≈20 min), defining the effective temporal window of cellular response. Time delay is expressed in frames (1 frame = 2 min). (E) Vector-field comparison of observed (blue) and predicted (red) acceleration across the field of view. Vector length indicates magnitude, and orientation indicates direction. Most cells are well captured by the model, although a few poorly predicted cells are also present. (F) Plot and heatmap showing the cosine similarity between observed and predicted acceleration vectors.

Fig 3A and 3B showed a quantitative agreement between the measured accelerations and those predicted by the inferred model. In Fig 3A–3B, we used an example cell in the test set, for which the trained model achieved the smallest prediction error. We then compared the shapes of the response functions obtained for the orthogonal and parallel directions relative to the wound edge (Fig 3C, 3D). Although a modest discrepancy was detected in the second spatial derivatives, no marked anisotropy emerged. The response function decayed to nearly zero within about 10 frames (∼20 min), suggesting that cells regulate their motion based on inputs within the past 20 minutes. This indicates that Eq (1) describes transient acceleration events associated with ERK wave propagation rather than persistent steady motion. To quantify the similarity between the observed and predicted acceleration values, we adopted the cosine-similarity index:


$$\text{cosine similarity}(a,b)=\frac{a\cdot b}{\left\|a\|\|b\right\|}$$

where *a* and *b* denote time courses of observed and predicted acceleration, respectively. Taken together, these results showed that the governing laws inferred from the time-series data accurately explained most cell movements; however, a small fraction of cells—scattered among otherwise well-predicted neighbors—seemed to obey different rules that the current model did not yet capture. The present model is deterministic. Stochasticity arising from measurement noise and intrinsic cell dynamics was not explicitly modeled, but was only reflected in the residual prediction errors (Fig 3F).

### Evaluation of response function properties of cellular units

**Fig 4:**
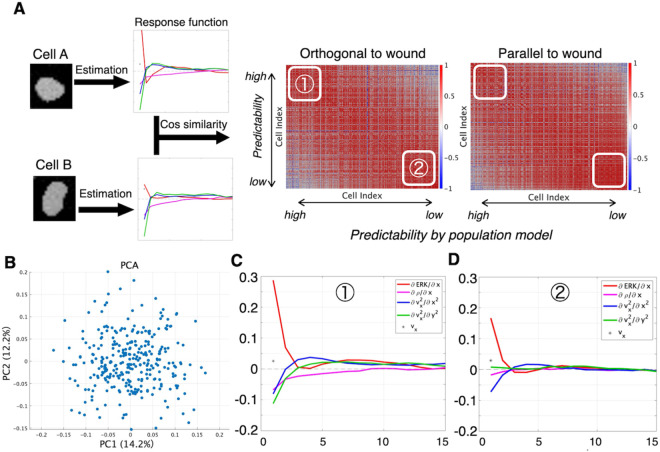
Analysis of cell-cell heterogeneity of response functions. (A) Heat map of pair-wise cosine similarity among response functions estimated from all 304 test-set cells individually. Cell index is defined as the ranking of cells in descending order of the correlation between their observed acceleration and prediction from the global response function. Columns and rows are ordered from left to right (top to bottom) by the prediction performance by the population-level response function from the training-set. Thus, well-predicted cells appear toward the upper left. (B) PCA of the test-cell response functions. Each point represents one cell, summarizing variability in the response patterns. (C) Mean orthogonal-to-wound response functions for the 20 cells (7 % of the test set) whose accelerations were predicted most accurately by the training-set population-level model. (D) Mean orthogonal-to-wound response functions for the 20 cells with the poorest prediction accuracy.

To elucidate the above-mentioned “rule heterogeneity,” we estimated a distinct response function for every single cell in the test population and assessed the pairwise similarity between cells (Fig 4). To quantify the similarity between a pair of response functions, we adopted the cosine-similarity index. For this calculation, response functions of each cell were represented as an array, constructed by concatenating the response function to individual inputs. Fig 4A presented the cosine-similarity matrix of these cell-specific response functions. In the heat-map axes, cells were sorted by how accurately their accelerations were predicted by the population-level response function derived from the training data, arranged from best to worst. Test cells that were well predicted by the training-derived response function exhibited high mutual similarity, as did those that were poorly predicted. Nevertheless, the cell-specific response functions did not form discrete clusters; rather, they lay along a continuous spectrum (Fig 4A and S5 FigA). We subjected the individual response functions of all test cells to principal-component analysis (PCA) and confirmed the continuous nature (Fig 4B and S5 FigB). To further characterize the rule heterogeneity, we calculated response functions using two 20-cell subsets: one containing cells that the population-level model predicted accurately and another containing cells it predicted poorly. Fig 4C and 4D (and S5 FigC,D) showed the response functions for the orthogonal-to-wound accelerations, for the well-predicted and poorly-predicted groups, respectively. In the well-predicted group (Fig 4C), we again observed a biphasic response to the ERK gradient, echoing the behavior of the training population. Moreover, the viscous drag exerted a positive influence on acceleration after a slight temporal delay, representing the temporal structure of the chemical and mechanical inputs. On the other hand, in the poorly-predicted group (Fig 4D), the response functions remained qualitatively similar but were generally flatter and of lower amplitude. This profile implied that these cells either lacked a tight coupling between the signals and acceleration or operated under a highly stochastic motility regime. Yet, the high cosine similarity observed within this group indicated that its members still followed a common kinetic rule.

### Response function in part of wound-healing

**Fig 5:**
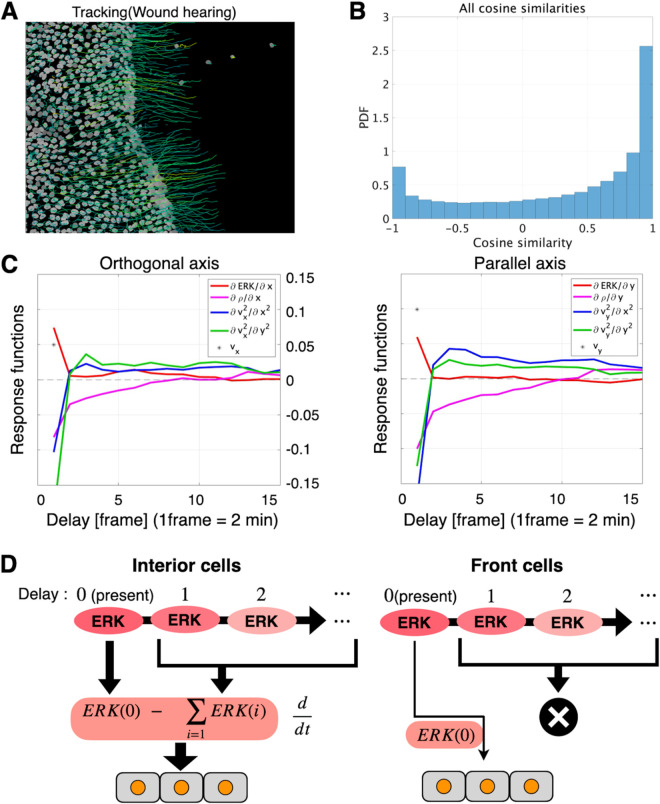
Front-edge cells rely on a motility rule that differs from that of interior cells. (A) Inference of response functions for front cells during wound healing. Left: cell trajectories (100 frames, 200 min) super-imposed on the FRET ratiometry image; the tracks point toward the wound. Isolated cells on the far right and tracks shorter than 100 frames were excluded. (B) Histogram of all cosine similarities. The orthogonal axis shows the probability density. (C) Response functions for acceleration orthogonal and parallel to the wound edge, respectively. Time delay is expressed in frames (1 frame = 2 min). In front cells, the response pattern to ERK gradient peaks at zero lag and then rapidly decays toward zero. (D) Schematic of ERK-gradient response rules in interior vs. front cells. For interior cells, the response function acting on the ERK gradient is differentiator-like, driving migration according to recent temporal changes rather than absolute levels. In front cells, the response function places virtually all weight at zero lag, indicating that migration depends mainly on the instantaneous ERK gradient.

Until now, our analysis had centred on cells embedded deep within the tissue, far from the wound margin. As exemplified by “leader cells” that emerged at the advancing edge, we reasoned that cells situated close to the wound might follow distinct motility rules. To test this hypothesis, we applied our inference framework to the cell cohort adjacent to the wound and calculated their response functions (Fig 5A, 5B). During wound closure, an ERK-activity wave propagated away from the lesion, creating a strong directional bias that drove cells predominantly along the orthogonal-to-wound axis. Surprisingly, however, the response functions for orthogonal and parallel motion were highly similar. Thus, even though the extracellular cues were highly anisotropic, the intrinsic law that governed cell movement remained effectively isotropic. A more detailed look at the response to the spatial gradient of ERK activity exposed a clear divergence between front and interior cells. In front cells, the response function peaked at the current ERK level and collapsed to near zero for all positive time lags (Fig 5C). Interior cells, by contrast, displayed a differentiator-like profile: the response peaked at zero lag, became negative, and then gradually returned to positive values (Fig 3C, 3D). This pattern implied that interior cells compared present ERK levels with their recent history when choosing how to move. Collectively, these findings showed that interior cells relied on changes in past ERK activity, whereas front cells near the wound based their behavior almost exclusively on instantaneous ERK signals (Fig 5D).

### Forward analysis of collective cell migration

**Fig 6:**
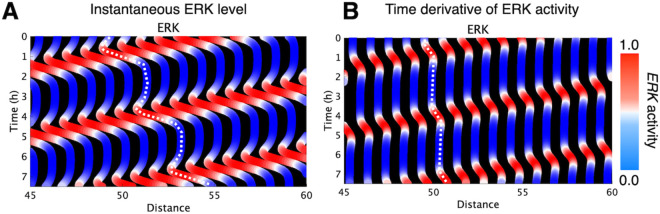
Forward analysis of collective cell migration. (A) The instantaneous ERK level (Eq 3 in previous study). (B) The time derivative of ERK activity. In this panel, the model implements the data-driven response function by replacing the dependence on the instantaneous ERK level with the time derivative of ERK activity, as inferred from the learned control law. Horizontal axis is cell scan position (Distance, 45-60) and orthogonal axis is elapsed time since experiment start (Time, 0-7 h). Color denotes the magnitude of each value. These panels visualize an ERK activity wave propagating from right to left over time. Parameter and setting of (A) are k = 2 (min^−2^), $\mu_{0}$ = 10 (min^−1^), *R*_0_ = 1/2, α = 1.5, β = 2.5, σ = 0.1 (min^−1^), sweeping velocity of illumination = 0.1 (min^−1^), width of illuminated area = 30, number of cells = 100. Parameter and setting of (B) are k = 2 (min^−2^), $\mu_{0}$ = 10 (min^−1^), *R*_0_ = 1/2, α = 10, β = 2.5, σ = 0.1 (min^−1^), sweeping velocity of illumination = 0.1 (min^−1^), width of illuminated area = 30, number of cells = 100. Where *k* is the spring constant between neighboring cells; *R*_0_ and $\mu_{0}$ are the basal cell radius and viscosity, respectively, modulated by ERK activity with amplitudes α(size) and β(viscosity); and σ is the ERK decay rate.

The differentiator-like response to ERK captured in our response function also serves as a useful guideline for constructing mechanistic models. To illustrate this point, we revisit the spring-mass model on which our regression framework was originally based (see Material and methods). Below we propose a modified spring-mass model that overcomes a known limitation of the original formulation. Our regression model, Eq (1), was developed by generalizing the spring-mass model, which treats cells as masses connected by springs, and by introducing history-dependent coefficients (see Material and methods). Based on the data-driven control law obtained in this study, we propose a modified spring-mass model that overcomes a known limitation of the original model. Fig 6 (and S6 Fig) showed a simulation time course of the original spring-mass model. Although the net motion of cells was opposite to the direction of ERK activity waves, our forward simulation revealed a transient movement of cells toward wave propagation upon wave arrival. This behavior was not observed in cultured epithelia. In the original model, cell radius changed in response to the instantaneous ERK level. Thus we modified the model so that cell radius instead changed in response to the time derivative of ERK activity, consistent with the temporal-differentiator behavior inferred from our data-driven response function. The full mathematical formulation of this revised model is provided in the Material and methods section. This modification eliminated the artifact of cells moving in the same direction as the ERK wave (Fig 6B and S6 FigB).

## Discussion

In this study, we developed an inverse-analysis framework that uncovered governing principles of cell motility grounded in quantitative biological evidence and leveraged them to predict cell movements with high fidelity. We applied the framework to simultaneously acquired time-series data of ERK activity and cell migration in cultured epithelial sheets obtained through FRET-based live imaging. The analysis revealed that the acceleration of individual cells could be quantitatively predicted from trajectories and ERK activities of local cells. The former reflects mechanical cues acting on each cell, such as local pushing and pulling forces, whereas the latter represents a tissue-wide spatiotemporal signal generated by the propagating ERK activity wave. Because our framework derived control laws directly from experimental time-series data, it provided a broadly applicable, data-driven route for extracting biologically meaningful regulatory rules from observed cellular dynamics.

The derived response functions revealed a distinctive temporal pattern for the spatial gradient of ERK activity. Specifically, the function exhibited a sharp positive peak at zero time lag, followed by a rapid decline that overshot into a transient negative phase. This biphasic shape implied that cells responded to ERK signals in a differentiator-like manner, generating mechanical reactions that were acutely sensitive to the current rate of change rather than to the absolute level of activity. The delayed negative component further indicated that cells recognized not only the initial rise but also the subsequent fall of ERK activity, suggesting an ability to anticipate—or at least account for—the dynamic profile of the ERK gradient arising from wave propagation. Eq (1) therefore applies primarily to transient phases of wound closure, when ERK gradients and mechanical cues fluctuate dynamically. As these inputs diminish at later stages, the right-hand side of the equation approaches zero, naturally yielding dv/dt ≈ 0 in the quasi-steady regime. The model thus encompasses both transient acceleration and steady motion within a unified framework. By contrast, the response functions for the density gradient and the second spatial derivatives of velocity field were smoother and more sustained, indicating that these mechanical cues acted on a slower timescale and became prominent after the sharp chemical trigger provided by ERK activity. Of note, the density-gradient response likely reflected an elastic counterforce, whereas the velocity-gradient response may capture a viscous one.

Such time-differentiator-like behavior has been reported to emerge intrinsically from negative feedback circuits across different cell types. As a classical example, in the *E. coli* signaling pathway, negative feedback mediated by receptor desensitization enables computation of the time derivative of extracellular chemical concentrations [30]. Another instance of temporal differentiation occurs in sensory neurons of C. elegans [31,32], where negative feedback through the GPCR-Ca^2+^-Calcineurin (TAX-6) pathway has been shown to generate transient responses proportional to the time derivative of stimulus intensity, as well as exact adaptation.

Together, these findings supported a two-step control scheme in which a rapid chemical stimulus initiated movement, followed by mechanical feedback that complemented and modulated the ensuing migration. How cells sense ERK activity gradients and which circuit elements mediate the resulting changes in acceleration remain unresolved questions and represent important future challenges. Direct perturbation of the ERK pathway is challenging to interpret because genetic or pharmacological manipulations on the ERK pathway often disrupt the ERK activity waves, thereby obscuring their downstream intracellular functions. Thus, it would be interesting to combine such perturbations with exogenous generation of ERK waves using optogenetic tools such as OptoRaf. A similar strategy could also be applied to mechanical regulators such as zonula occludens-1 (ZO-1), which also influence ERK waves.

In our results, the response functions governing cell migration were highly similar between the axes orthogonal and parallel to the wound, indicating that the intrinsic cellular responses were nearly isotropic. This suggests that the anisotropy of collective cell migration primarily arises from anisotropic external cues, namely, the absence of cells at the wound and the propagation of ERK activity waves from the wound edge. However, previous studies have reported single-cell level anisotropy in moving monolayer [12,29]. It would therefore be intriguing to incorporate such polarity information into future analyses. Specifically, instead of defining the axes of the response functions based on the global x-y coordinate system of the monolayer, one could define them along and perpendicular to each cell polarity. This approach may help reveal anisotropy that was otherwise averaged out in our current analysis.

When we compared response-function similarity at the single-cell level, we found that cells that were accurately captured by the population-average model shared similarly shaped response functions—and likewise for cells that the population model predicted poorly. Interestingly, similarity analysis across all cells showed no discrete clusters; rather, the response functions formed a continuous spectrum. In other systems, however, truly discrete clusters might exist, and our approach should have been able to uncover such hidden cell types. In those cases, relying on a single population-average model would have been inadequate, making it important to extend our framework with hierarchical modeling. The cell-cell heterogeneity may arise from intrinsic sources such as stochastic fluctuations in ERK signaling or cell-cycle stage, as well as extrinsic factors, including local variations in mechanical tension. These factors can also interact, as exemplified by force-dependent modulation of the cell cycle [33]. Together, these considerations highlight the importance of incorporating multiscale heterogeneity into future models.

At the wound front, we uncovered control rules that differed from those operating in interior cells, most strikingly for the chemical cue represented by the spatial derivative of ERK activity. Wound-front cells lacked the biphasic response to the ERK gradient observed in interior cells and instead exhibited a single positive-peaked response. In other words, these cells did not behave as differentiators that read the rate of change of ERK gradient; they accelerated in proportion to the current ERK level. Consistent with this, sustained ERK activation was frequently observed at wound margins, suggesting that front cells employed motion-control rules tuned to a persistent chemical signal. The density gradient, by contrast, exerted an overall inhibitory influence, suppressing movement toward the high-density interior. This difference likely reflects distinct schemes of chemical communication operating over different length scales. As suggested in studies of chemotaxis [34], establishing a stable long-range gradient is inherently difficult; instead, propagating ERK waves provides an effective means of transmitting chemical cues to interior cells. In contrast, cells at the leading edge can rely simply on the ERK activity gradient emanating from the wound edge to sustain their forward migration. In the current formulation, we focused on the short-timescale causal influence of ERK activity on cell motion and did not include potential feedback from cell motion and cell-cell signaling to ERK dynamics. Due to this limitation, our model cannot predict or generate the spatiotemporal patterns of ERK activity waves themselves. However, previous studies have reported that ERK activity can be modulated by mechanical tension [19] and E-cadherin-mediated contact signaling. Incorporating such bidirectional coupling between ERK signaling and cell mechanics represents an important future extension of our framework.

Here, we discuss the limitations of our current framework. The regression model we presently employed assumed linearity, leaving potential nonlinear effects largely unexamined. Expanding the model to incorporate nonlinear interactions would have made it possible to capture higher-order crosstalk among additional MAPK family members and mechanosensory pathways, yielding a more comprehensive view of the control architecture governing cell motility. For example, the activation of ERK signaling can exhibit threshold-like or saturating behaviors, potentially generating switch-like transitions or plateaus in motile responses [35]. Moreover, although bypassed in the present study, the intercellular propagation of ERK activity is also inherently nonlinear, and its detailed mechanisms remain controversial. These biological nonlinearities pose substantial technical challenges from an inverse-analysis perspective. Linear convolution kernels would need to be replaced by nonlinear model classes, such as polynomial expansions or neural network-based response functions; however, the associated modeling and optimization strategies remain active areas of research [36]. Such approaches provide a promising means to incorporate thresholding, saturation, and cooperative responses while maintaining interpretability of the inferred control laws. In addition, cell collectives likely adapted to non-stationary environments by dynamically re-weighting chemical and mechanical cues that guide their behavioral choices. The culture-based framework developed in this study did not yet accommodate such flexible signal weighting. A recent machine-learning framework for physiological data from outdoor-reared animals offers a useful approach for addressing this issue. In that framework, meteorological data are integrated with physiological measurements, allowing one to infer how fish weigh different environmental signals such as temperature [37]. Incorporating a similar strategy—estimating model-parameter weights directly from environmental factors and cellular time-series data—into our regression pipeline could have enabled us to derive context-dependent control laws in vivo from experimental data.

The machine-learning of response functions not only allowed us to predict cell migration but also informed targeted improvements to an existing mathematical model (Fig 6). Moreover, these data-driven insights furnished a principled foundation for probing the specific molecular pathways that implement the inferred control behaviors, thereby offering a valuable framework for guiding subsequent experimental validation.

This study advanced both the mechanistic understanding of multicellular dynamics and the methodology of data-driven modeling. By inferring governing laws directly from time-series imaging, we quantitatively uncovered a mechanochemical control law that connected single-cell mechanobiology to tissue-scale behavior. Grounded in a general regression framework, the method could be transferred from MDCK monolayers to a broad array of multicellular systems, opened new avenues for quantitative analyses of collective motion in biomedical and engineering contexts. We believe these results laid a foundation for extracting governing principles from complex phenomena that had resisted traditional bottom-up modeling.

## Material and methods

### Image analysis

We outlined the image-analysis workflow used to extract single-cell features for training our machine-learning model. ERK activity waves during scratch-wound healing were visualized previously with a Förster resonance energy transfer (FRET) biosensor and time-lapse microscopy [22]. From these movies we extracted cell trajectories with TrackMate, an ImageJ/Fiji plug-in that links detected cells across frames via cost-minimizing assignment [38–40]. The resulting cell coordinates were processed with a second-order Savitzky-Golay filter (window = 11 frames) to obtain first- and second-order temporal derivatives, that is, cell velocities and accelerations, respectively. In addition, we obtained spatial derivatives of the velocity field by least-squares fitting a quadratic polynomial to the velocity vectors of neighboring cells within a radius *r* around each cell.

FRET and CFP channels were first smoothed with a Gaussian kernel (σ=1.0px) to attenuate high-frequency noise. After subtracting background fluorescence, we computed pixel-wise FRET/CFP ratio images for every time point. The ratio value at each cell centroid served as a single-cell measure of ERK activity. Analogous to the velocity analysis, ERK activity values of neighboring cells within a radius r(=10) were least-squares fitted to a quadratic surface, and analytical differentiation of the fitted coefficients yielded the local spatial gradient of ERK activity.

Cell density fields were reconstructed for every frame by kernel density estimation, superimposing a two-dimensional Gaussian kernel (σ=25px) at each cell position. Spatial gradients of the resulting density maps were calculated with the same local quadratic fitting procedure described above.

### Mathematical model

We previously proposed a mathematical model of epithelial cell migration driven by propagation waves of ERK activity [22,28]:


$$\frac{dv_{i}}{dt}=-\mu_{i}v_{i}-k[(R_{i+1}+R_{i})-(x_{i+1}-x_{i})]+k[(R_{i}+R_{i-1})-(x_{i}-x_{i-1})]+\eta (v_{i+1}-2v_{i}+v_{i-1})$$

In this model, each cell *i* was characterized by several parameters: its velocity $v_{i}$, viscosity η, radius *R*_*i*_, and friction coefficient $\mu_{i}$. Here, *x*_*i*_ denotes the position of the *i*-th cell along the one-dimensional array. The elasticity of the interaction was described by a spring constant *k*. The cell elasticity *k* and tissue viscosity η are assumed to be common to all cells. We subsequently derived a coarse-grained hydrodynamic description of the particle-based model to facilitate analytical analysis. In our previous work [28], we derived a one-dimensional continuum version of the model and heuristically extended it to two dimensions. In the present study, we provide a more explicit derivation of the two-dimensional continuum description and introduce additional tissue viscosity terms for capturing realistic epithelial mechanics(see Supplementary Text S1 for the derivation).

$$\frac{Dv_{x}}{Dt}≃\overset{ERK-dependent\;friction}{\overbrace{-\mu_{0}(1-\beta ERK)v}}-\overset{ERKactivity}{\overbrace{\frac{2\alpha kR_{0}}{\rho }\frac{\partial ERK}{\partial x}}}-\overset{density}{\overbrace{\frac{k}{\rho^{3}}\frac{\partial \rho }{\partial x}}}+\overset{tissue\;viscosity}{\overbrace{\frac{\eta }{\rho^{2}}\frac{\partial^{2}v_{x}}{\partial x^{2}}+\frac{\eta }{\rho^{2}}\frac{\partial^{2}v_{x}}{\partial y^{2}}}}$$ (2)

In this model, $v_{x}$ and $v_{y}$ represented velocities in the orthogonal and parallel directions relative to the wound axis, respectively; μ was the friction coefficient; β was a scaling factor for ERK-dependent friction; *ERK* denoted the molecular activity of ERK; α was a scaling factor relating ERK activity to cell size; *k* was the elastic (spring) constant; *R*_0_ was the basal cell radius; ρ was the density; and η was the viscosity coefficient. Note that Eq (2) is valid only under high-cell-density conditions. Its applicability to MDCK monolayers has been empirically demonstrated [28]. This equation indicated that the acceleration of cells could be approximated as a linear combination of mechanical parameters and spatial derivatives of ERK activity. Note that the derivation introduced the term $\partial_{xx}v_{x}$ and $\partial_{yy}v_{x}$, which was absent in the model proposed in our previous work. This term was expected to be effective for capturing the spatiotemporal patterns of cell velocity observed in the data. By substituting the coefficient of each term with *R*_0_–*R*_4_, we obtained Eq (1) in the main text.

### 1D Spring model

We introduced the mathematical model used in Fig 6, which was composed of a one-dimensional (1D) array of cells. Since each cell adhered to its neighbors, cells mechanically interacted through repulsive and attractive forces. To represent these mechanical interactions, we assumed a system in which particles were connected by springs. Here, each particle represented the centroid position of a cell, and the springs represented the elastic properties of the cells, including membrane, cytoskeleton, and adhesion. Accordingly, the dynamics of the position of the *i*-th cell *x*_*i*_ were described as follows:


$$\frac{dx_{i}}{dt}=v_{i}\;\frac{dv_{i}}{dt}=-\mu_{i}v_{i}+k\left(x_{i+1}-x_{i})-k(R_{i}+R_{i+1}\right)-k\left(x_{i}-x_{i-1})+k(R_{i}+R_{i-1}\right)$$

where $v_{i}$ denoted the velocity of the *i*-th cell, and *k* represented the spring constant of the cell. At the boundaries of the particle array, the end particles were coupled to only one neighboring particle.

In our previous model used in Fig 6B, *R*_*i*_ and $\mu_{i}$ were modulated by the ERK activity as follows:


$$R_{i}=R_{0}(1+\alpha ERK_{i})\;\mu_{i}=\mu_{0}\exp(-\beta ERK_{i})$$

Here, *ERK*_*i*_ denoted the ERK activity of the *i*-th cell, while *R*_0_ and $\mu_{0}$ represented the basal cell radius and basal viscosity, respectively. Parameters α and β described the effects of ERK activity on cell size and viscosity, respectively. The ERK-dependent expansion (*R*_*i*_) has been validated by optogenetic activation of ERK [29]. It is also reasonable to expect that the friction coefficient (μ) is ERK-dependent, since ERK phosphorylates FAK. However, the effect currently lacks direct experimental validation.

On the other hand, in Fig 6C, we modified the following update rule for the basal cell radius:


$$R_{i}=R_{0}(1+\alpha \cdot \partial ERK_{i}/\partial t)$$

We modeled optogenetically induced ERK activation, which was expressed as:


$$\frac{dERK_{i}}{dt}=-\sigma ERK_{i}+L_{i}$$

where σ and *L*_*i*_ denoted the decay rate of ERK activity and the light intensity applied to the *i*-th cell, respectively. *L*_*i*_ = 1 for illuminated cells while = 0 otherwise. The sweeping velocity of illumination was set to 0.1 (min^−1^), and the width of the illuminated area was 30.

### Dataset preparation

We separately cropped non-overlapping regions from the entire field of view of the imaging data, which were used to prepare the training and test sets. To exclude cells that moved into or out of the field of view or appeared through cell division, we limited the analysis to trajectories lasting at least 400 frames. This procedure removed unstable trajectories and extremely short time series that would otherwise have added noise during training. For the same reason, cell trajectories containing missing values were discarded. The resulting training set comprised 398 cell trajectories, each beginning at the first frame and extending for at least 400 frames. The test set contained 303 trajectories processed in the same way. No data augmentation was applied in this study.

### Machine learning

We extended the continuum model (Eq (2)) to incorporate the time-series profiles of the mechano-chemical inputs, thereby increasing its quantitative ability to fit real-world data. Past information was incorporated by constructing, for each explanatory variable, a temporal convolution matrix. Specifically, for the response variable, cell acceleration, we assumed the following convolutional model:

$$\frac{dv}{dt}=w_{0}-w_{1}x_{1}+\sum_{i=2}^{5}\int_{0}^{t}w_{i}(t-\tau )x_{i}(\tau )d\tau$$ (3)

In the regression model, the explanatory variables included a bias term *w*_0_, the velocity component $x_{1}=v_{x}$, the spatial gradient of ERK activity $x_{2}=\partial ERK/\partial x$, the spatial gradient of cell density $x_{3}=\partial \rho /\partial x$, and the second spatial derivatives of velocity, $x_{4}=\partial^{2}v_{x}/\partial x^{2}$ and $x_{5}=\partial^{2}v_{x}/\partial y^{2}$. Here, τ denoted the temporal lag (time shift), and *w*_*i*_(*t* − τ) was the weight for the *i*-th predictor at that lag. Because cell acceleration is the time derivative of velocity, allowing an arbitrary temporal function for velocity would have let the model approximate acceleration almost exactly, thereby masking the contributions of the other predictors. For this reason, velocity was included in the model only at the current time point, with no temporal history. After temporal discretization, the convolutional formulation became a multivariate linear regression problem that we fit by ridge regression, with regularization strength controlled by the parameter λ. Let λ denote the regularization strength, i.e., the ridge penalty is $\lambda \|\theta {\|}^{2}$ where $\|\theta {\|}^{2}$ is the L2 norm of the parameter vector. The regularization parameter was optimized by five-fold cross-validation, yielding an optimal value of λ=784.760 (S3 Fig). In addition, to verify the stability of the characteristic shapes of the response functions, we confirmed that the functions inferred from randomly sampled data subsets exhibited consistent profiles (S4 Fig). The length of the temporal history considered was limited to 20 frames, chosen from the inter-predictor cross-correlation structure observed in the training data (Fig 2B).

## Supporting information

S1 Fig Live imaging of ERK-induced collective cell migration (a) Time-lapse FRET imaging of ERK activity during wound healing in an epithelial MDCK monolayer expressing a nuclear-localized ERK biosensor. Snapshots are shown 1 h (upper) and 9 h (lower) after scratching. Red and blue denote high and low ERK activity, respectively; the cytosol is not visualized because the biosensor is confined to the nucleus. (b) Kymograph of ERK activity in the band region outlined by the white box in (a). (c) Schematic of ERK-mediated collective migration. As an ERK activity wave travels, cells migrate in the opposite direction.
(TIFF)

S2 Fig Scatter plot and correlation of training data (a) Scatter plot of the input variables supplied to the machine-learning model that predicts cell acceleration orthogonal to the wound edge. (b)Heat map of the input features shown in (a). A weak positive correlation is observed between cell acceleration and the spatial gradient of ERK activity, whereas cell velocity shows weak negative correlations with both of its corresponding second spatial derivatives. (c) Scatter plot of the input variables for predicting cell acceleration parallel to the wound edge. (d) Heat map of the input features shown in (c). Similar weak correlations are observed, with acceleration positively linked to ERK gradients and velocity negatively linked to its second derivatives.
(TIFF)

S3 Figk-fold cross-validation.Results of k-fold cross-validation used to optimize the regularization parameter λ. Only the training set was employed. The left panel reports the cross-validation error over a broad search range ($10^{-4}\leq \lambda \leq 10^{4}$). To locate the minimum, cross-validation was repeated within a narrower neighborhood ($10^{-1}\leq \lambda \leq 10^{3.75}$), yielding an optimal value of λ=784.7600.(TIFF)

S4 FigGroup k-fold cross-validation.To assess the stability of the fitted regularization parameter λ, we performed grouped k-fold cross-validation (*k* = 5) and compared the response function profiles obtained from each fold. The analysis was conducted for the four parameters that incorporated time delay. All five folds yielded nearly identical response functions, confirming the robustness and stability of the fitted λ.(TIFF)

S5 Fig Analysis of cell-cell heterogeneity of response functions. (a) Heat map of pair-wise cosine similarity among response functions estimated from all 304 test-set cells individually. Columns and rows are ordered from left to right (top to bottom) by the prediction performance by the population-level response function from the training-set. Thus, well-predicted cells appear toward the upper left. (b) PCA of the test-cell response functions. Each point represents one cell, summarizing variability in the response patterns. (c) Mean parallel-to-wound response functions for the 20 cells (7% of the test set) whose accelerations were predicted most accurately by the training-set population-level model. (d) Mean parallel-to-wound response functions for the 20 cells with the poorest prediction accuracy.
(TIFF)

S6 FigNumerical simulation of one dimensional particle and spring model.Horizontal axis: cell scan position (Distance, 45-60); orthogonal axis: elapsed time since the start of the experiment (Time, 0-7 h). Colors indicate the magnitude of each quantity. Top left: Heat-map of ERK activity, illustrating a wave that propagates from right to left over time. Top right: Temporal derivative of ERK activity; warm colors denote wave initiation, whereas cool colors correspond to regions where the wave subsides. Bottom left: Spatial gradient of the temporal derivative, capturing heterogeneity in the driving forces at the leading and trailing edges of the ERK wave. Bottom right: Spatial derivative of ERK activity, highlighting the steepness of concentration changes at the wave front and rear and revealing local features such as wave width and shape. (a)The instantaneous ERK level (Eq 3 in previous study [22]) (b)The time derivative of ERK activity A comparison of the top-left panels shows that quantifying the rate of change in ERK activity mitigates the apparent forward-propagation artifact observed in the previous forward analysis. Parameter and setting of (a) are k = 2 (min^−2^), $\mu_{0}$ = 10 (min^−1^), *R*_0_ = 1/2, α = 1.5, β = 2.5, σ = 0.1 (min^−1^), sweeping velocity of illumination = 0.1 (min^−1^), width of illuminated area = 30, number of cells = 100. Parameter and setting of (b) are k = 2 (min^−2^), $\mu_{0}$ = 10 (min^−1^), *R*_0_ = 1/2, α = 10, β = 2.5, σ = 0.1 (min^−1^), sweeping velocity of illumination = 0.1 (min^−1^), width of illuminated area = 30, number of cells = 100. Where *k* is the spring constant between neighboring cells; *R*_0_ and $\mu_{0}$ are the basal cell radius and viscosity, respectively, modulated by ERK activity with amplitudes α(size) and β(viscosity); and σ is the ERK decay rate.(TIFF)

S1 Video ERK Activity in Wound Healing of MDCK Cells. Collective cell migration and ERK Activity in Wound Healing of MDCK Cells. MDCK cells were scratched on the right side, and started collective migration to wound edge. Red and blue indicate high and low ERK activity, respectively.
(MP4)

S1 Text Derivation of a two-dimensional continuum model of ERK-dependent collective cell migration. This text provides the detailed mathematical derivation that connects the discrete particle-based model of epithelial cell motion to a two-dimensional continuum formulation. The derivation incorporates ERK-dependent friction, cell-size modulation, and tissue viscosity, forming the theoretical basis for Eq (2) in the main text.
(PDF)
