# Supplementary figures and images for: Inverse modeling unveils governing law of mechano-chemical dynamics of epithelial migration
Source: PLoS Comput Biol. 2025 Dec 29;21(12):e1013854. doi: 10.1371/journal.pcbi.1013854 (PMC12782426; doi:10.1371/journal.pcbi.1013854)

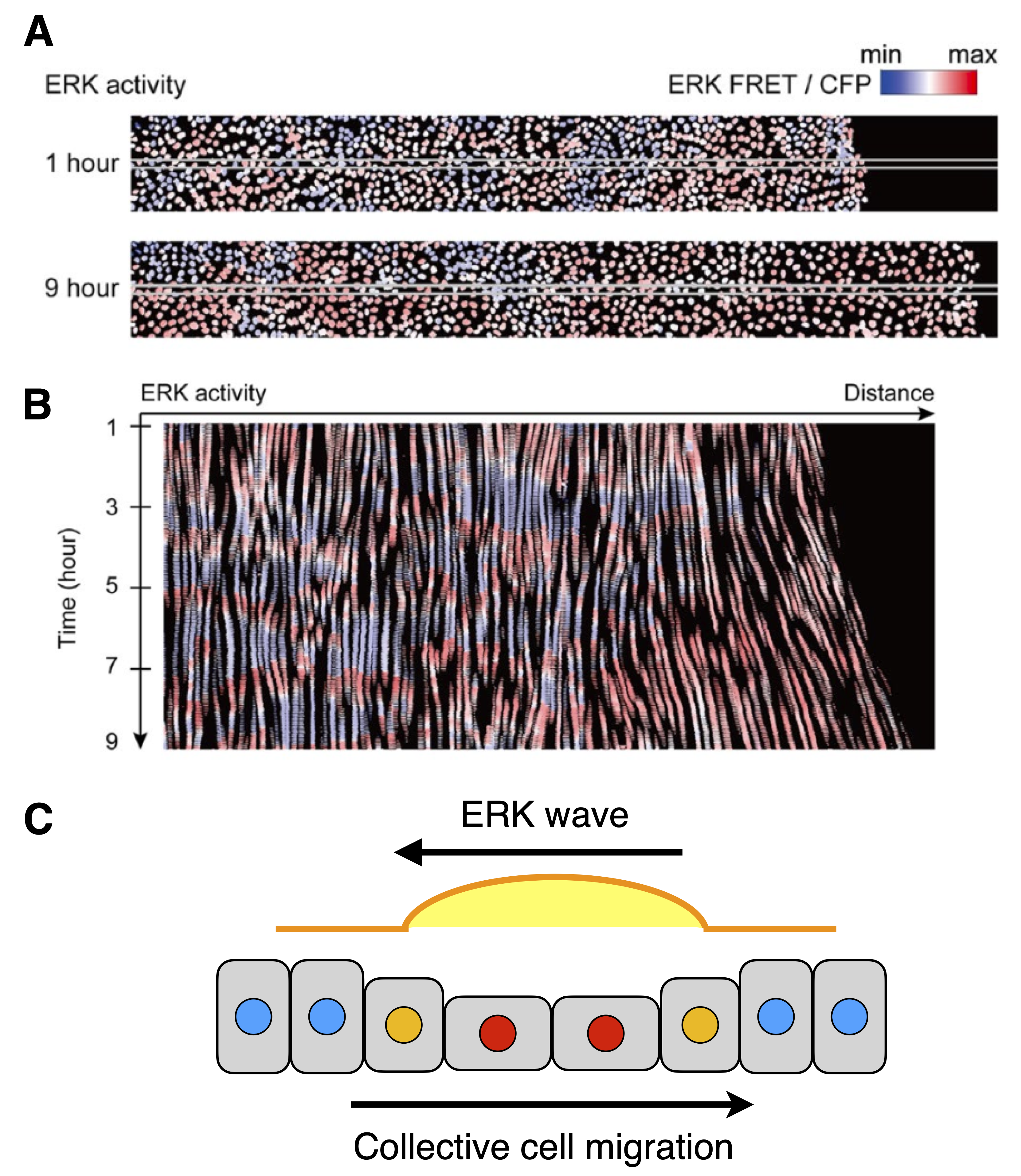

Supplement: S1 Fig — (a) Time-lapse FRET imaging of ERK activity during wound healing in an epithelial MDCK monolayer expressing a nuclear-localized ERK biosensor. Snapshots are shown 1 h (upper) and 9 h (lower) after scratching. Red and blue denote high and low ERK activity, respectively; the cytosol is not visualized because the biosensor is confined to the nucleus. (b) Kymograph of ERK activity in the band region outlined by the white box in (a). (c) Schematic of ERK-mediated collective migration. As an ERK activity wave travels, cells migrate in the opposite direction. (TIFF) [file pcbi.1013854.s001.tiff]

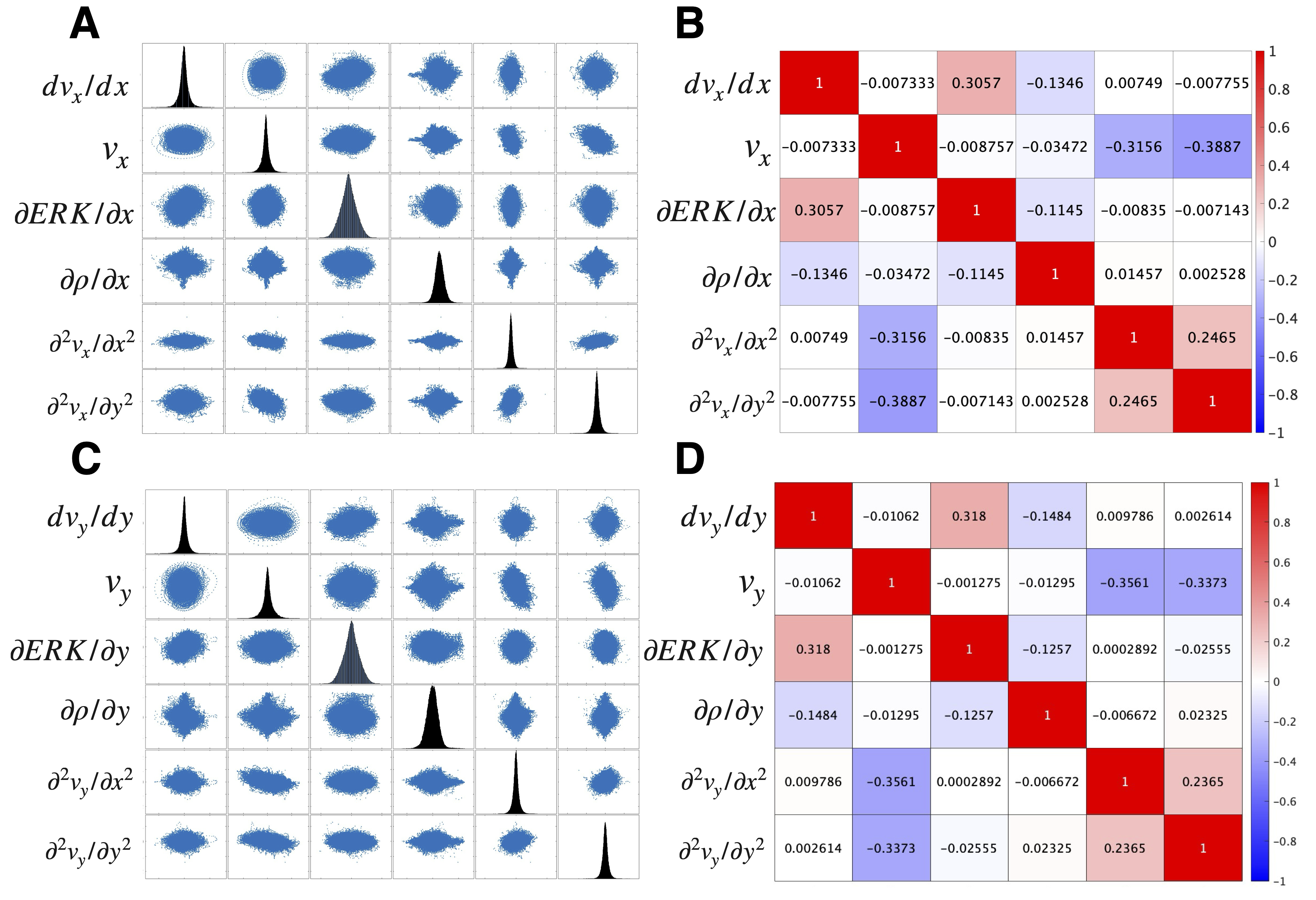

Supplement: S2 Fig — (a) Scatter plot of the input variables supplied to the machine-learning model that predicts cell acceleration orthogonal to the wound edge. (b)Heat map of the input features shown in (a). A weak positive correlation is observed between cell acceleration and the spatial gradient of ERK activity, whereas cell velocity shows weak negative correlations with both of its corresponding second spatial derivatives. (c) Scatter plot of the input variables for predicting cell acceleration parallel to the wound edge. (d) Heat map of the input features shown in (c). Similar weak correlations are observed, with acceleration positively linked to ERK gradients and velocity negatively linked to its second derivatives. (TIFF) [file pcbi.1013854.s002.tiff]

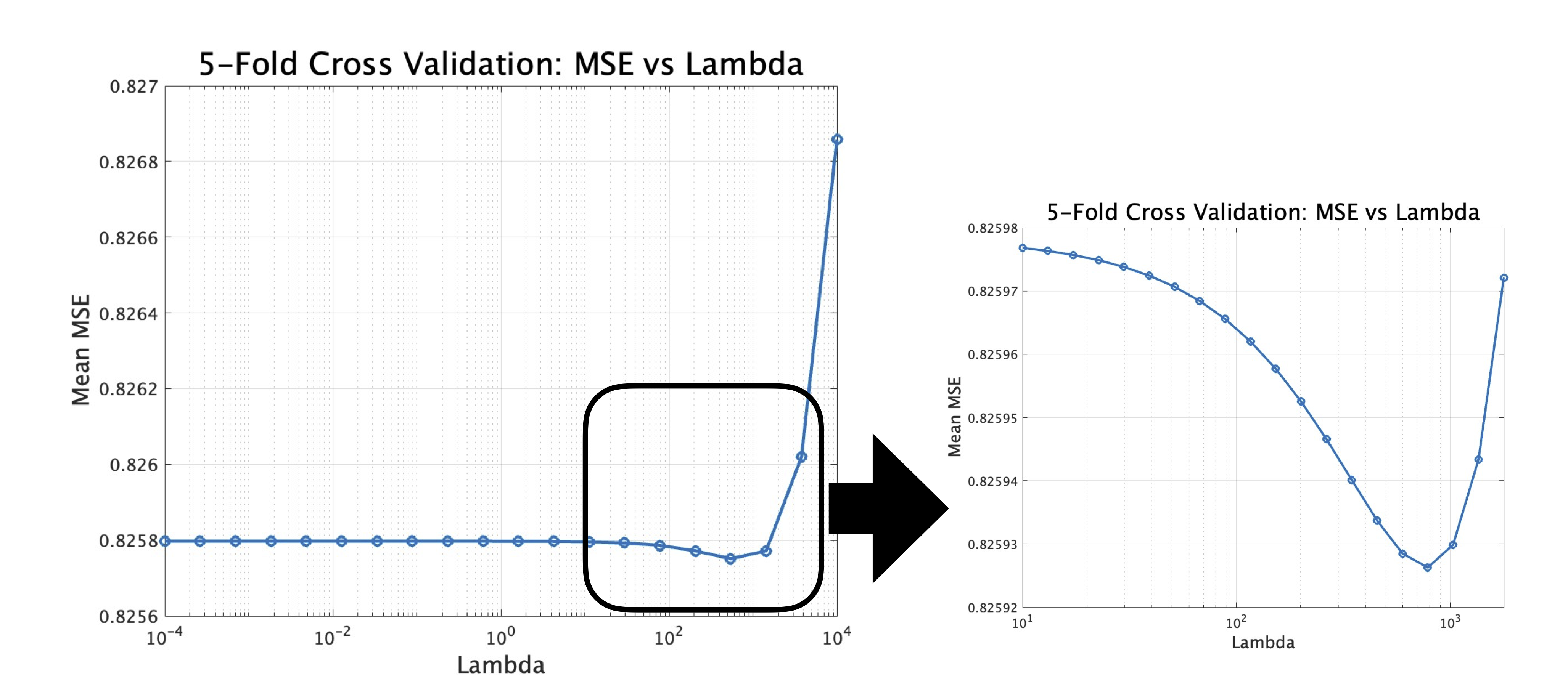

Supplement: S3 Fig — Results of k-fold cross-validation used to optimize the regularization parameter λ. Only the training set was employed. The left panel reports the cross-validation error over a broad search range (10−4≤λ≤104). To locate the minimum, cross-validation was repeated within a narrower neighborhood (10−1≤λ≤103.75), yielding an optimal value of λ=784.7600. (TIFF) [file pcbi.1013854.s003.tiff]

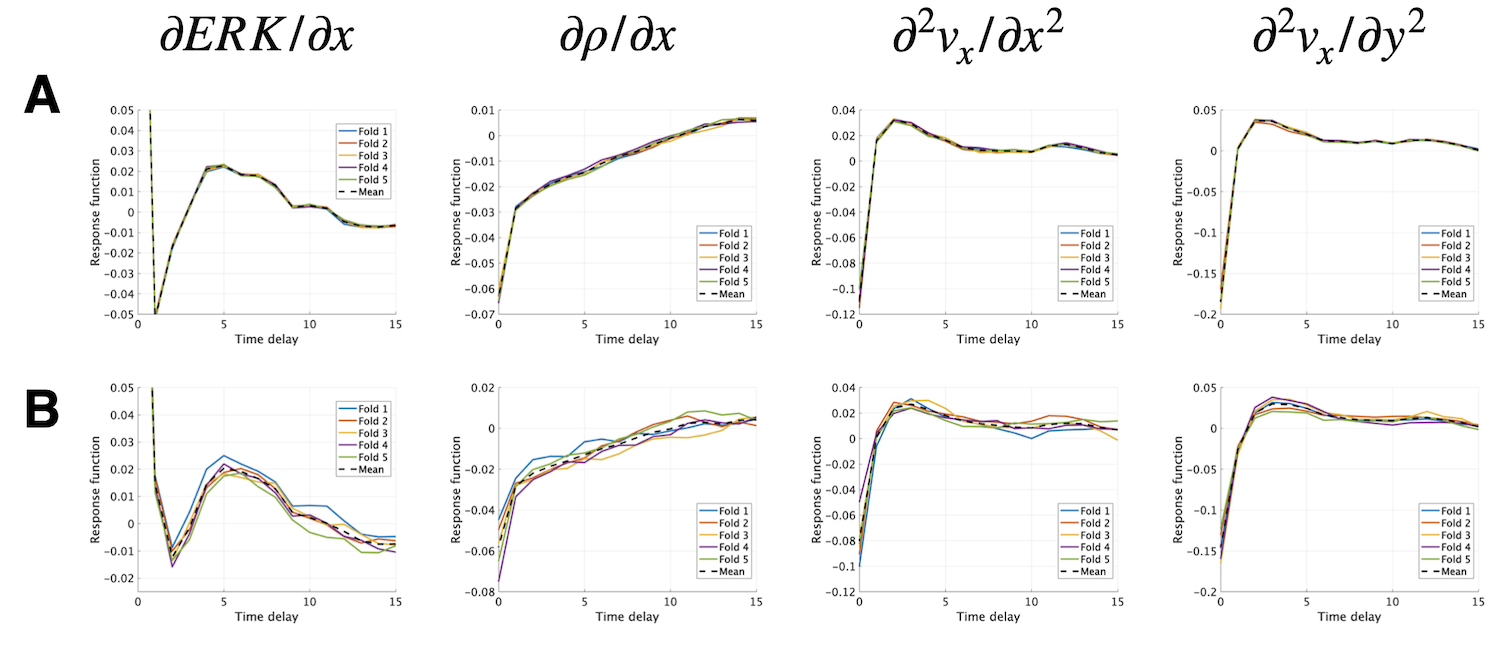

Supplement: S4 Fig — To assess the stability of the fitted regularization parameter λ, we performed grouped k-fold cross-validation (k = 5) and compared the response function profiles obtained from each fold. The analysis was conducted for the four parameters that incorporated time delay. All five folds yielded nearly identical response functions, confirming the robustness and stability of the fitted λ. (TIFF) [file pcbi.1013854.s004.tiff]

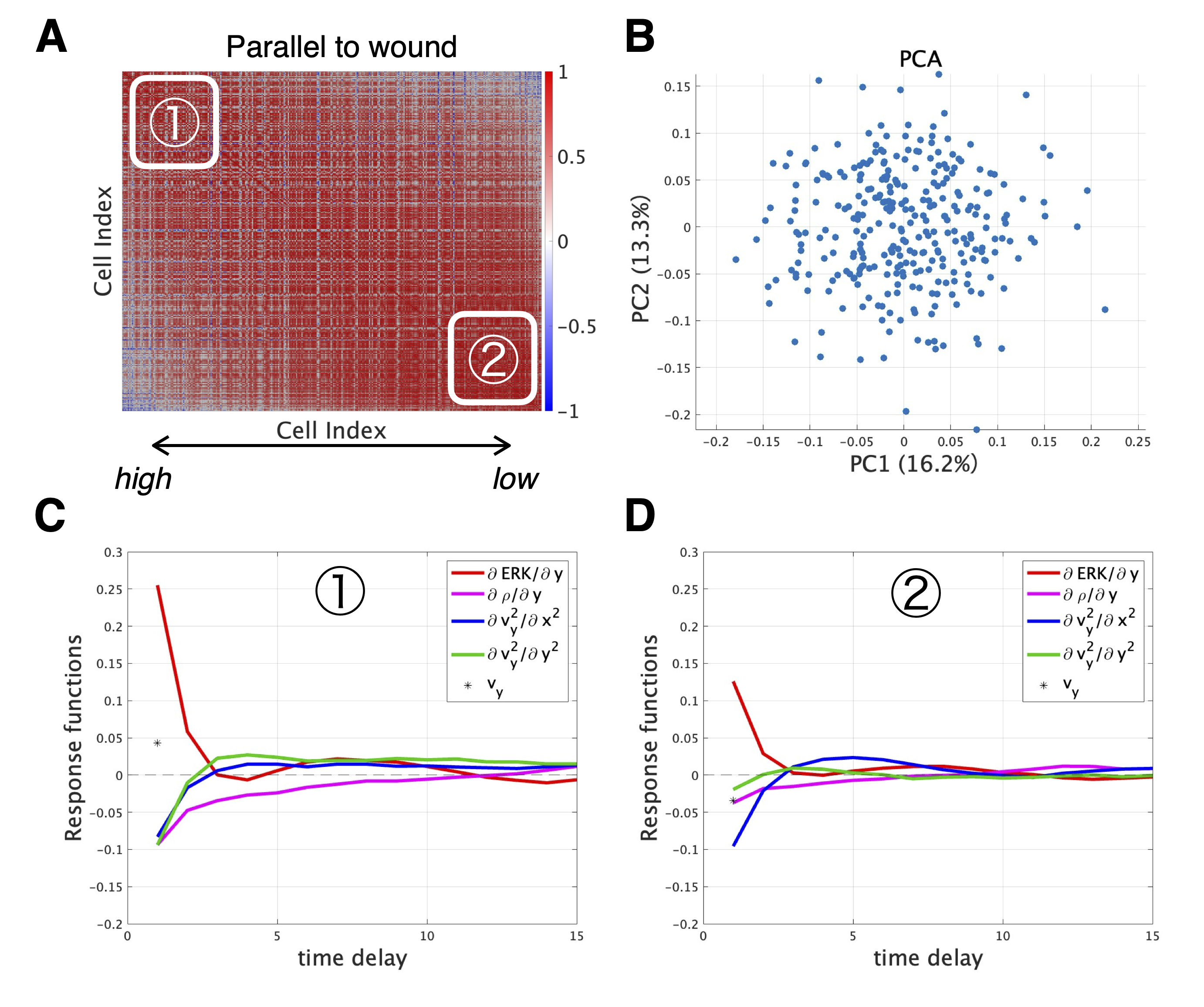

Supplement: S5 Fig — (a) Heat map of pair-wise cosine similarity among response functions estimated from all 304 test-set cells individually. Columns and rows are ordered from left to right (top to bottom) by the prediction performance by the population-level response function from the training-set. Thus, well-predicted cells appear toward the upper left. (b) PCA of the test-cell response functions. Each point represents one cell, summarizing variability in the response patterns. (c) Mean parallel-to-wound response functions for the 20 cells (7% of the test set) whose accelerations were predicted most accurately by the training-set population-level model. (d) Mean parallel-to-wound response functions for the 20 cells with the poorest prediction accuracy. (TIFF) [file pcbi.1013854.s005.tiff]

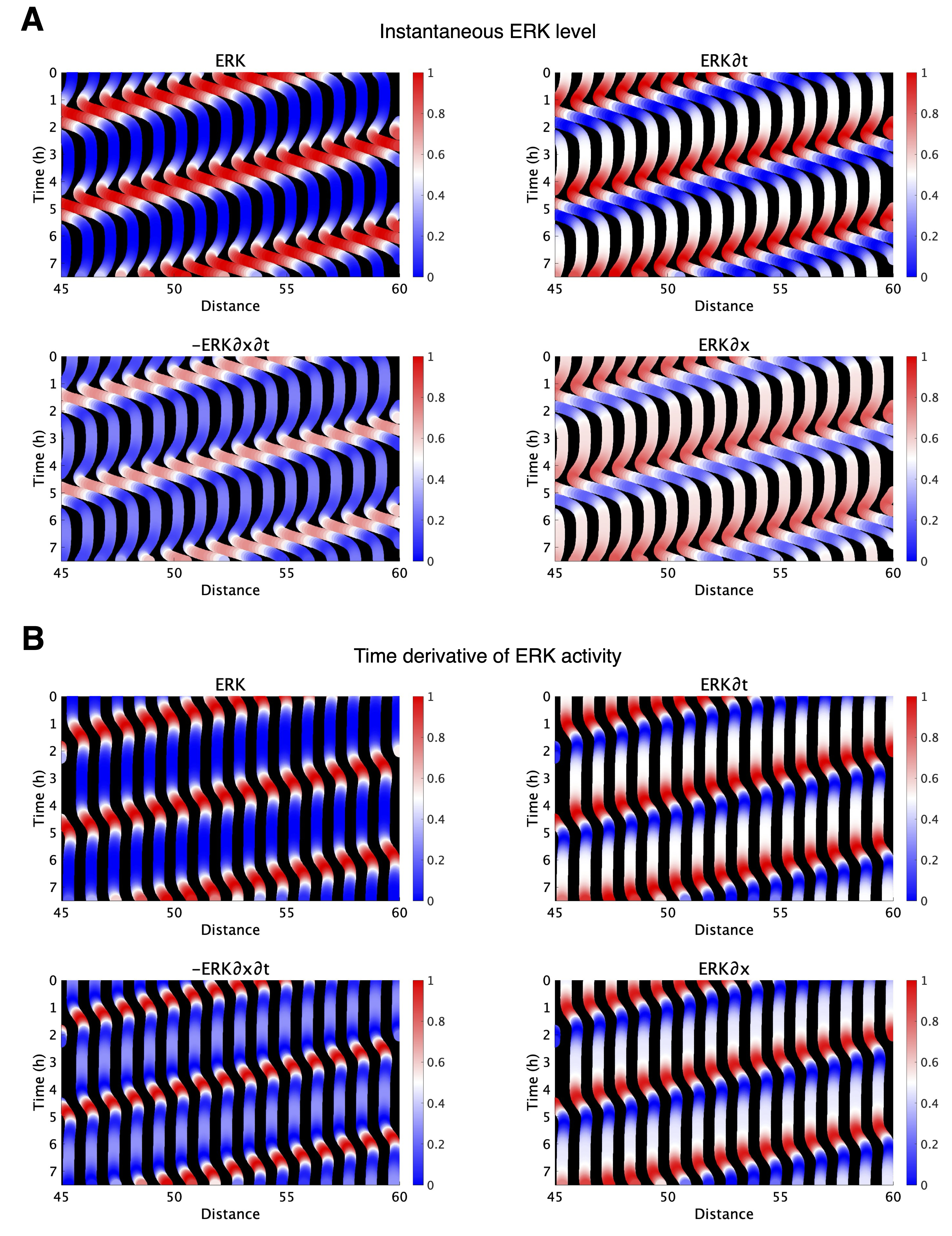

Supplement: S6 Fig — Horizontal axis: cell scan position (Distance, 45-60); orthogonal axis: elapsed time since the start of the experiment (Time, 0-7 h). Colors indicate the magnitude of each quantity. Top left: Heat-map of ERK activity, illustrating a wave that propagates from right to left over time. Top right: Temporal derivative of ERK activity; warm colors denote wave initiation, whereas cool colors correspond to regions where the wave subsides. Bottom left: Spatial gradient of the temporal derivative, capturing heterogeneity in the driving forces at the leading and trailing edges of the ERK wave. Bottom right: Spatial derivative of ERK activity, highlighting the steepness of concentration changes at the wave front and rear and revealing local features such as wave width and shape. (a)The instantaneous ERK level (Eq 3 in previous study [22]) (b)The time derivative of ERK activity A comparison of the top-left panels shows that quantifying the rate of change in ERK activity mitigates the apparent forward-propagation artifact observed in the previous forward analysis. Parameter and setting of (a) are k = 2 (min−2), μ0 = 10 (min−1), R0 = 1/2, α = 1.5, β = 2.5, σ = 0.1 (min−1), sweeping velocity of illumination = 0.1 (min−1), width of illuminated area = 30, number of cells = 100. Parameter and setting of (b) are k = 2 (min−2), μ0 = 10 (min−1), R0 = 1/2, α = 10, β = 2.5, σ = 0.1 (min−1), sweeping velocity of illumination = 0.1 (min−1), width of illuminated area = 30, number of cells = 100. Where k is the spring constant between neighboring cells; R0 and μ0 are the basal cell radius and viscosity, respectively, modulated by ERK activity with amplitudes α(size) and β(viscosity); and σ is the ERK decay rate. (TIFF) [file pcbi.1013854.s006.tiff]
