# Supplementary material for: Inverse modeling unveils governing law of mechano-chemical dynamics of epithelial migration
Source: PLoS Comput Biol. 2025 Dec 29;21(12):e1013854. doi: 10.1371/journal.pcbi.1013854 (PMC12782426; doi:10.1371/journal.pcbi.1013854)
Supplement: S1 Text — This text provides the detailed mathematical derivation that connects the discrete particle-based model of epithelial cell motion to a two-dimensional continuum formulation. The derivation incorporates ERK-dependent friction, cell-size modulation, and tissue viscosity, forming the theoretical basis for Eq (2) in the main text. (PDF) [file pcbi.1013854.s008.pdf]

S1 Text. Derivation of a two-dimensional continuum model of ERK-dependent collective cell migration

We describe in detail the discrete-to-continuum approximation procedure used in this study. Our approach closely follows previous works [1, 2]. As a starting point, we assume the following model for each of the two coordinate directions, x and y:

$$\frac{dv_i}{dt} = -\mu_i v_i - k(R_{i-1} - R_{i+1}) + k(x_{i+1} - 2x_i + x_{i-1}) + \eta(v_{i+1} - 2v_i + v_{i-1}) \quad \text{(S1)}$$

In this model, each cell i was characterized by several parameters: its velocity v_i, spring constant between neighboring cells k, viscosity η, radius R_i, and friction coefficient μ_i. Here, x_i denotes the position of the i-th cell along the one-dimensional array. Note that the equation holds for x- and y-directions. This model assumes cell mobility and cell area on the substrate to depend on the ERK activity. First, the ERK activity promotes cell mobility, which is effectively represented by reduction in cell-substrate friction as:

$$\mu_i v_i = \mu_0(1 - \beta \cdot ERK_i)v_i \quad \text{(S2)}$$

where β is the coefficient for ERK-dependent friction and ERK_i is the ERK activity in the i-th cell. Second, the ERK activity increases cell radius R:

$$R_i = R_0(1 + \alpha ERK_i) \quad \text{(S3)}$$

There is a biological basis for defining R and μ as ERK-dependent. It has been reported that ERK activation reduces the contractile force of MDCK cells and increases their cell area [3]. In addition, ERK-mediated phosphorylation of FAK (Focal Adhesion Kinase) induces dynamic changes in focal adhesions, effectively altering the cellular frictional properties. We approximate $(R_{i-1} - R_{i+1})$ as

$$\begin{aligned} R_{i\pm1} &= R \pm \frac{\Delta}{2}\partial_x R + \mathcal{O}(\Delta^2) \\ &= R_0\left[1 + \alpha ERK \pm \frac{\alpha R_0}{2}\partial_x ERK\right] + \cdots \end{aligned}$$

then

$$\begin{aligned} R_{i+1} - R_{i-1} &\simeq \alpha R_0 \Delta \partial_x ERK \\ &= \frac{\alpha R_0}{\rho}\partial_x ERK \quad \text{(S4)} \end{aligned}$$

Here, Δ denotes the lattice spacing (mean inter-cell distance) and is related to the local density by $\Delta = \frac{1}{\rho}$. In the discrete model, a physical quantity associated with the i-th cell, $f(x_i)$, is defined only at discrete cell positions, whereas in the continuous description we introduced a smooth field $f(x)(x \in R)$. Correspondingly, we consider a continuous interpolation of the cell index and approximate $x_{i+1} - 2x_i + x_{i-1}$ as

$$x_{i+1} - 2x_i + x_{x-1} \approx \frac{x(i+\Delta) - 2x(i) + x(i-\Delta)}{\Delta^2}$$

By performing a Taylor expansion, $x(i+\Delta) = x(i) + \Delta \frac{\partial x}{\partial i} + \frac{\Delta^2}{2}\frac{\partial^2 x}{\partial i^2} + \cdots$, we further approximate the central difference as

$$
\begin{aligned}
x_{i+1} - 2x_i + x_{i-1} &\approx \frac{x(i+\Delta) - 2x(i) + x(i-\Delta)}{\Delta^2} \\
&= \frac{\partial^2 x}{\partial i^2} + \mathcal{O}(\Delta^4) \\
&\approx \frac{1}{\rho^2}\partial_{xx}x - \frac{1}{\rho^3}\partial_x\rho\partial_x x + \mathcal{O}(\Delta^4) \\
&\approx -\frac{1}{\rho^3}\partial_x\rho \qquad \text{(S5)}
\end{aligned}
$$

Here, we assumed that physical quantities vary slowly between neighboring cells so that $(\partial i)^n \sim \text{Order}(\Delta^n)$. We also used $\partial_i = (\partial x/\partial i)\partial_x = \Delta\partial_x$. Similarly, for the tissue viscosity term,

$$
\begin{aligned}
\eta(v_{i+1} - 2v_i + v_{i-1}) &\approx \frac{v(i+\Delta) - 2v(i) + v(i-\Delta)}{\Delta^2} \\
&= \eta\left\{\frac{\partial^2 v}{\partial i^2} + \mathcal{O}(\Delta^4)\right\} \\
&\approx \eta\left(\frac{1}{\rho^2}\partial_{xx}v_x - \frac{1}{\rho^3}\partial_x\rho\partial_x v_x + \mathcal{O}(\Delta^4)\right) \\
&\approx \eta\left(\frac{1}{\rho^2}\partial_{xx}v_x - \frac{1}{\rho^3}\partial_x\rho\partial_x v_x\right)
\end{aligned}
$$

As in our previous attempt, we used separate one-dimensional models for the x- and y-axes for simplicity. Then the resulting viscous term for the x-axis depends only on the velocity gradient along the x direction. However, in a real two-dimensional tissue, it is reasonable to expect contributions from velocity gradients along the y direction as well. To account for this, we supplement the tissue viscosity term as follows. Therefore, the tissue viscosity term becomes

$$\frac{\eta}{\rho^2}(\partial_{xx}v_x + \partial_{yy}v_x) - \frac{\eta}{\rho^3}(\partial_x\rho\partial_x v_x + \partial_y\rho\partial_y v_y) \qquad \text{(S6)}$$

Finally, expressing the time derivative of particle location in Eulerian coordinates gives

$$\frac{d}{dt} \to \frac{D}{Dt} \equiv \partial_t + \mathbf{v}\cdot\nabla \qquad \text{(S7)}$$

Taken together(S1 - S7), we obtain:

$$\frac{Dv_x}{Dt} = -\mu_0(1-\beta ERK)v - \frac{2\alpha k R_0}{\rho}\partial_x ERK - \frac{k}{\rho^3}\partial_x\rho + \frac{\eta}{\rho^2}(\partial_{xx}v_x + \partial_{yy}v_x) - \frac{\eta}{\rho^3}(\partial_x\rho\partial_x v_x + \partial_y\rho\partial_y v_y)$$

An analogous expression holds for by swapping x and y. In our regression analysis, we omit the final term for simplicity because (i) the velocity Laplacian alone can represent tissue viscosity, and (ii) retaining the final term could lead

to overfitting and induce numerical stability. Thus, we obtain Equation (2) in the main text.

$$\frac{Dv_x}{Dt} \simeq -\mu_0(1-\beta ERK)v - \frac{2\alpha k R_0}{\rho}\partial_x ERK - \frac{k}{\rho^3}\partial_x \rho + \frac{\eta}{\rho^2}\partial_{xx}v_x + \frac{\eta}{\rho^2}\partial_{yy}v_x \quad (2)$$

References

[1] Y Asakura, Y Kondo, K Aoki, N Honda. Hierarchical modeling of mechano-chemical dynamics of epithelial sheets across cells and tissue. Scientific reports. 2021;11:4069. doi:10.1038/s41598-021-83396-6.

[2] P J Murray, C M Edwards, M J Tindall, P K Maini. Classifying general nonlinear force laws in cell-based models via the continuum limit. Phys Rev E. 2012;85:021921. doi:10.1103/PhysRevE.85.021921.

[3] T Fukuyama, H Ebata, A Yamamoto, R Ienaga, Y Kondo, M Tanaka, et al. Why epithelial cells collectively move against a traveling signal wave. Soft Matter. 2025;21:7881-94. doi:10.1039/D5SM00403a.
